# Does exchange arthroplasty of an infected shoulder prosthesis provide better eradication rate and better functional outcome, compared to a permanent spacer or resection arthroplasty? a systematic review

**DOI:** 10.1186/s12891-016-0901-6

**Published:** 2016-02-01

**Authors:** D. A. George, A. Volpin, S. Scarponi, F. S. Haddad, C. L. Romanò

**Affiliations:** Department of Trauma and Orthopaedics, University College London Hospitals, 235 Euston Road, London, NW1 2BU UK; Centre for Reconstructive Surgery and Osteoarticular Infections, Orthopaedic Research Institute Galeazzi, Milan, Italy

**Keywords:** Eradication rate, Functional outcome, Periprosthetic shoulder infection, Single-stage, Two-stage, Resection arthroplasty, Permanent spacer

## Abstract

**Background:**

The best surgical modality for treating chronic periprosthetic shoulder infections has not been established, with a lack of randomised comparative studies. This systematic review compares the infection eradication rate and functional outcomes after single- or two-stage shoulder exchange arthroplasty, to permanent spacer implant or resection arthroplasty.

**Methods:**

Full-text papers and those with an abstract in English published from January 2000 to June 2014, identified through international databases, such as EMBASE and PubMed, were reviewed. Those reporting the success rate of infection eradication after a single-stage exchange, two-stage exchange, resection arthroplasty or permanent spacer implant, with a minimum follow-up of 6 months and sample size of 5 patients were included.

**Results:**

Eight original articles reporting the results after resection arthroplasty (*n* = 83), 6 on single-stage exchange (*n* = 75), 13 on two-stage exchange (*n* = 142) and 8 papers on permanent spacer (*n* = 68) were included.

The average infection eradication rate was 86.7 % at a mean follow-up of 39.8 months (SD 20.8) after resection arthroplasty, 94.7 % at 46.8 months (SD 17.6) after a single-stage exchange, 90.8 % at 37.9 months (SD 12.8) after two-stage exchange, and 95.6 % at 31.0 months (SD 9.8) following a permanent spacer implant. The difference was not statistically significant (*p = 0.650*).

Regarding functional outcome, patients treated with single-stage exchange had statistically significant better postoperative Constant scores (mean 51, SD 13) than patients undergoing a two-stage exchange (mean 44, SD 9), resection arthroplasty (mean 32, SD 7) or a permanent spacer implant (mean 31, SD 9) (*p = 0.029*). However, when considering studies comparing pre- and post-operative Constant scores, the difference was not statistically significant.

**Conclusion:**

This systematic review failed to demonstrate a clear difference in infection eradication and functional improvement between all four treatment modalities for established periprosthetic shoulder infection. The relatively low number of patients and the methodological limitations of the studies available point out the need for well designed multi-center trials to further assess the best treatment option of peri-prosthetic shoulder infection.

## Background

Periprosthetic joint infection (PJI) is a devastating complication following hemiarthroplasty and total arthroplasty of the shoulder. It complicates 0 to 3.9 % of primary anatomical shoulder arthroplasty [1–3], and 2 to 18.8 % of reverse shoulder arthroplasty [4–6]. The surgical management of such cases is varied and includes either a single- or two-stage exchange arthroplasty [7, 8], resection arthroplasty with or without a permanent spacer [9, 10], arthroscopic lavage or open debridement with implant retention [11], arthrodesis [12] or amputation [13].

With no clear management guidelines, there are ongoing discussions regarding the advantages of each treatment strategy. The aim of treatment is to eradicate infection and prevent recurrence, with the challenge of optimising function of the joint in light of potential soft tissue compromise, bone loss, and large dead space in the subacromial region [14].

Unlike PJI following total hip and knee arthroplasty, the two-staged exchange is not considered the ‘gold-standard’ of treatment, with numerous prospective and retrospective cohort studies demonstrating favorable results of other treatment options [7, 9, 15, 16], however no definitive comparison of their outcome has been analysed in a randomized control study.

In this comprehensive systematic review of the literature, we aim to determine which, if any, treatment option provides better infection eradication rates, as well as enabling high functional outcomes for established periprosthetic shoulder infections. We hypothesized that single- or two-stage revision surgery results in better infection eradication rate and functional outcome than other treatment modalities.

## Methods

Studies reporting the eradication rate of infection and/or functional outcomes following revision procedures to the shoulder as a result of periprosthetic infections, published from January 2000 to June 2014, were reviewed.

The criteria for inclusion of studies were as follows: (a) Studies with an abstract or written fully in English; (b) Studies reporting the results of infection eradication following a single-stage arthroplasty, two-stage arthroplasty, and resection arthroplasty with or without a permanent spacer (excluding debridement with implant retention, arthrodesis and amputation); (c) Studies reporting the functional results following a single-stage arthroplasty, two-stage arthroplasty, and resection arthroplasty with or without a permanent spacer; (d) Studies relating to delayed or chronic (6 weeks or later) stages of disease; (e) Study design was either a randomised controlled trial; comparative prospective study; prospective study with historical controls; prospective case series with no comparison group; comparative retrospective study; retrospective study with historical control group; or retrospective study with no control group; (f) Only the longest follow-up and largest patient series was included if more than one paper by the same author(s) was retrieved and if the patient cohort was deemed to be similar and the follow-ups were found to overlap; (g) The study cohort had to include 5 or more cases, even if treated with different surgical procedures; (h) The follow-up had to be of a minimum 6 months; (i) If reporting infection recurrence rate following treatment, the following variables had to be reported: number of patients treated; type of treatment; number of recurrent infections; and (j) If reporting functional outcome following the treatment, the following variables had to be reported: number of patients treated; type of treatment; pre- and/or post-operative functional scores (including Neer and Constant scores), as well as other measures such as range of motion and activity of daily living. No studies were excluded based upon the indication of the primary procedure (i.e. proximal humeral fracture, osteoarthritis, rotator cuff arthropathy, rheumatoid arthritis, or avascular necrosis).

International databases were searched systematically as previously described by Romano et al. [17]. Databases included: EMBASE; PubMed/Medline; Medline Daily Update; Medline In-Process and other non-indexed citations; Google Scholar; SCOPUS; CINAHL; Cochrane Central Register of Controlled Trials and Cochrane Database of Systematic Reviews; NHS Health Technology Assessment; http://www.google.com; and http://www.yahoo.com. Keywords were used alone or in various combinations to identify relevant papers: shoulder; infection; arthroplasty; prosthesis; shoulder replacement; prosthetic joint infection; periprosthetic joint infection; exchange arthroplasty; one-stage; single-stage; two-stage; resection arthroplasty; and permanent spacer.

**Fig. 1:**
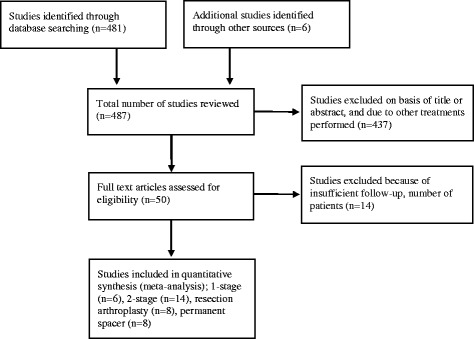
PRISMA flow diagram of exclusion of papers during the systematic review process [Adopted from: Moher D, Liberati A, Tetzlaff J, Altman DG, The PRISMA Group. Preferred Reporting Items for Systematic Reviews and Meta-Analyses: The PRISMA Statement. PLoS Med 2009;6(6):e1000097]

Figure 1 illustrates the systematic exclusion of papers in this review. Previously published criteria to assess the quality of studies in systematic reviews, was utilized in this paper [18–20] however the quality score was not used as an exclusion criterion. This included evidence of: (a) Patient cohort demographics (age and sex, indications for index shoulder procedure, isolated pathogens); (b) Description of the treatment modality (indications, length of antibiotic therapy, interim period length between stages, implants types); (c) Reported outcomes (frequency of recurrent infections, number of patients lost to follow-up).

Four investigators, DAG, AV, SS and CLR, searched and reviewed the literature independently, and then compared and combined their lists to complete the literature search. Any discrepancies were solved by reclassification as mutually agreed. Initial inclusion was based upon the studies title and abstract, but the latter stages of the review process excluded papers based on the inclusion criteria.

Statistical analysis was performed using *t*-tests when analyzing two treatment modalities directly, and ANOVA (one-way analysis of variance) to compare all four treatment groups. In addition Fisher’s Exact test was used to compare bivariate data of means. A *p*-value of <0.05 was deemed to be statistically significant.

## Results

The systematic review included eight original articles relating to resection arthroplasty (*n* = 83), 6 on single-stage exchange (*n* = 75), 13 on two-stage exchange (*n* = 142) and 8 papers on permanent spacer (*n* = 68).

All studies reported on a remarkably limited series of patients and presented material was often diverse, reporting a variety of treatment modalities. The range in the number of cases was from 2 to 35 for a single-stage exchange, 2 to 19 for a two-stage exchange, 5 to 21 for resection arthroplasty, and 1 to 15 for permanent spacers. The mean duration of follow-up was 46.8 months (standard deviation (SD) 17.6) for single-stage exchange, 37.9 months (SD 12.8) after two-stage exchange, 39.8 months (SD 20.8) after resection arthroplasty, and 31.0 months (SD 9.8) after permanent spacer implant (Table 1).

**Table 1:** Infection eradication rates after revision arthroplasty for periprosthetic shoulder infection

Author	Year	Number of patients	Infections eradicated	Rate (%)	Follow-up (months)		
					Min	Max	Mean
Resection arthroplasty							
Sperling [27]	2001	21	15	71.4	33.6	157.2	78
Coste [21]	2004	10	7	70.0	12	96	32
Maynou [28]	2006	10	10	100.0			44
Braman [2]	2006	7	7	100.0	12	42	20.4
Rispoli [29]	2007	13	13	100.0			8.3
Verhelst [9]	2011	11	9	81.8			46.4
Weber [10]	2011	5	5	100.0	14.4	120	48
Romanò [30]	2012	6	6	100.0	24	98	41.1
Total *Mean (SD)*		83	72	86.7	*19.2 (9.4)*	*102.6 (41.9)*	*39.8 (20.8)*
Single-stage arthroplasty							
Sperling [27]	2001	2	1	50	9	120	57.6
Coste [21]	2004	3	3	100	12	96	32.0
Ince [16]	2005	14	14	100	13	159	69.6
Cuff [7]	2007	10	10	100	25	66	43.0
Beekman [14]	2010	11	10	90.9	12	36	22.4
Klatte [31]	2013	35	33	94.3	13.2	159	56.4
Total *Mean (SD)*		75	71	94.7	*14.0 (5.6)*	*106 (49.8)*	*46.8 (17.6)*
Two-stage arthroplasty							
Sperling [27]	2001	3	3	100	26.4	106.8	57.6
Seitz [32]	2002	8	8	100	36	96	56
Jerosh [3]	2003	8	8	100	6	30	
Coste [21]	2004	10	6	60	12	96	32
Cuff [7]	2007	12	12	100	25	66	43
Strickland [8]	2008	19	12	63.2	24	80	35
Themistocleous [33]	2008	2	2	100	15	26	22
Coffey [34]	2010	12	12	100	10	29	18.3
Stine [35]	2010	15	15	100			27.6
Jawa [36]	2011	15	14	93.3	12	69	27.6
Sabesan [37]	2011	17	16	94.1	22	80	46.2
Weber [10]	2011	4	4	100	14.4	120	48
Romanò [30]	2012	17	17	100	24	98	41.1
Total *Mean (SD)*		142	129	90.8	*18.9 (8.7)*	*74.7 (31.8)*	*37.9 (12.8)*
Permanent spacer							
Jerosh [3]	2003	2	2	100	6	30	18
Coste [21]	2004	1	1	100	12	96	32
Themistocleous [33]	2008	9	9	100	15	26	22
Coffey [34]	2010	4	4	100	16	25	19.25
Stine [35]	2010	15	15	100			28.8
Jawa [36]	2011	12	10	83.3	12	69	27.6
Verhelst [9]	2011	10	10	100			46.4
Romanò [30]	2012	15	14	93.3	24	98	41.1
Total *Mean (SD)*		68	65	95.6	*14.2 (5.9)*	*57.3 (34.8)*	*31 (9.8)*

The database search retrieved no randomized controlled trials with the majority of papers describing a retrospective case series without a control group. The quality of included studies is shown in Table 2, with a mean 74.89 % (SD 11.82) of all 11 variables reported in the studies (min 54.55 %, max 90.91 %). Specifically for a single-stage exchange 78.79 % (SD 13.69) of the variables were reported, 74.13 % (SD 8.17) reported for a two-stage, 64.94 % (SD 8.18) reported for resection arthroplasty, and 73.73 % (SD 9.72) reported for a permanent spacer. Only 8 studies (38.10 %) gave a detailed description of their criteria for selecting each treatment modality. Other relevant variables such length of antibiotic therapy (47.62 %) and duration of interim period between the two stages of a two-staged exchange (46.15 %) were poorly reported.

**Table 2:** Quality of papers included in the analysis

Quality indicators	Resection arthroplasty (*n* = 7, %)		Single-stage (*n* = 6, %)		Two-stage (*n* = 13, %)		Permanent spacer (*n* = 8, %)	
Age	7	*100*	6	*100*	13	*100*	8	*100*
Gender	7	*100*	6	*100*	13	*100*	8	*100*
Indication for primary shoulder arthroplasty	7	*100*	5	*83.3*	10	*76.9*	5	*62.5*
Isolated pathogen	6	*85.7*	6	*100*	13	*100*	8	*100*
Indication for type of revision procedure	3	*42.9*	3	*50*	4	*30.8*	3	*37.5*
Length of antibiotic therapy	1	*14.3*	4	*66.7*	6	*46.2*	4	*50*
Duration of interim period between stages	-		-		6	*46.2*	3	*37.5*
Implant type used at exchange arthroplasty	-		5	*83.3*	7	*53.8*	4	*50*
Number of reinfections	7	*100*	6	*100*	13	*100*	8	*100*
Functional outcome scores	5	*71.4*	5	*83.3*	9	*69.2*	6	*75*
Number of patients lost to follow-up	7	*100*	6	*100*	12	*92.3*	7	*87.5*
Mean (*%,* SD)	64.94	8.18	78.79	13.69	74.13	8.17	73.73	9.72

The highest rate of infection eradication was observed following the use of a permanent spacer (95.6 %), then a single-stage exchange (94.7 %), two-stage exchange (90.8 %) and resection arthroplasty (86.7 %) (Table 1). However this difference was not statistically significant (*p = 0.650*) when comparing all treatment options together, or when compared individually to one another (Table 3).

**Table 3:** Infection eradication rates between the different treatment modalities

Revision arthroplasty		Infection status at follow-up (n, %)				Significance (*p*-value)*
		Eradicated		Recurrence		
Permanent Spacer		65	*95.6*	3	*4.4*	
	Resection arthroplasty	72	*86.7*	11	*13.3*	*0.0897*
	Single-stage	71	*94.7*	4	*5.3*	*1.0000*
	Two-stage	129	*90.8*	13	*9.15*	*0.2771*
Single-stage		71	*94.7*	4	*5.3*	
	Resection arthroplasty	72	*86.7*	11	*13.3*	*0.1080*
	Two-stage	129	*90.8*	13	*9.15*	*0.4291*
Two-stage		129	*90.8*	13	*9.15*	
	Resection arthroplasty	72	*86.7*	11	*13.3*	*0.3743*

* Statistically significant if *p* < 0.05

Regarding functional outcome, it should be noted not all papers reported this data and a variety of evaluation measures were used; the Constant-Murley score was the most commonly and consistently reported scoring system (Table 4). However, very few studies provided pre- and post-operative functional scores and this makes analysis of data particularly challenging (Table 5). In the only three studies that reported pre-operative Constant score, this was, on average, significantly better in patients undergoing single-stage revision (score 37, SD 3), compared to those treated with a two-stage procedure (score 16, SD 1) (*p < 0.0001)*. Coste et al. [21] reported also pre-operative Constant score in 10 patients undergoing resection arthroplasty and in only 3 patients treated with a permanent spacer, showing a better pre-operative function in patients treated with single-stage exchange compared to both the other treatment modalities.

**Table 4:** Functional scoring systems used by included studies (*n* = 17)

Functional scores	Frequency	Percentage
Constant-Murley	11	*64.70*
UCLA Shoulder Score	2	*11.76*
Simple Shoulder Test (SST)	6	*35.29*
American Shoulder and Elbow Surgeons (ASES)	3	*17.65*
Neer-Score	3	*17.65*
Pennsylvania (PENN) shoulder score	2	*11.76*
Disabilities of the Arm, Shoulder and Hand (DASH)	6	*35.29*

**Table 5:** Functional outcome following revision surgery for shoulder periprosthetic infection; comparison of mean follow-up, and constant scores for each treatment modality

Author	Year	Number of patients	Follow-up (months)			Constant score		
			Min	Max	Mean	Pre-op	Post-op	Difference
Resection arthroplasty								
Coste [21]	2004	10	12	96	32	16	30	14
Weber [10]	2011	5	14	120	48	-	33	
Debeer [38]	2006	7			9	-	26	
Verhelst [9]	2011	11	17	101	46	-	46	
Romano [30]	2012	6	24	98	41	-	32	
Ghijselings [39]	2013	6	18	97	49	-	28	
Total *Mean (SD)*		45	*17 (5)*	*102 (10)*	*38 (15)*		*32 (7)*	
Single-stage arthroplasty								
Coste [21]	2004	3	12	96	32	35	66	31
Ince [16]	2005	9	13	159	70	-	34	
Beekman [14]	2010	11	12	36	22	39	51	12
Klattle [31]	2013	35	13	159	56	-	51	
Total *Mean (SD)*		58	*13 (1)*	*113 (59)*	*45 (22)*	*37 (3)*	*51 (13)*	*21 (14)*
Two-stage arthroplasty								
Jerosch [3]	2003	8	6	30		-	48	
Coste [21]	2004	10	12	96	32	15	35	20
Weber [10]	2004	4	14	120	48	-	40	
Coffey [34]	2010	12	10	29	18	16	57	41
Romano [30]	2012	17	24	98	41	-	38	
Total *Mean (SD)*		51	*13 (7)*	*75 (42)*	*35 (13)*	*16 (1)*	*44 (9)*	*31 (15)*
Permanent spacer								
Coste [21]	2004	3	12	96	32	26	38	12
Romano [30]	2012	15	24	98	41	-	34	
Ghijselings [39]	2013	4	67	87	78	-	21	
Total *Mean (SD)*		22	34 (29)	94 (6)	50 (24)		*31 (9)*	

The poorly reported pre-operative Constant scores and the different baseline in different cohorts of patients should be taken into consideration when comparing post-operative function. In fact, considering post-operative Constant scores, patients treated with single-stage exchange (mean score 51, SD 13) or a two-stage revision (44, SD 9) seem to perform better than those undergoing resection arthroplasty (32, SD 7) or a permanent spacer implant (31, SD 9). However, when considering the average difference between pre- and post-operative Constant score, no statistical difference can be seen any more (Table 6).

**Table 6:** Post-operative functional outcome scores (and change of scores) between the different treatment modalities

Revision arthroplasty		Post-operative constant score		Significance (*p*-value)*
		Score (mean)	SD	
Permanent spacer		31.0	8.89	
	Resection arthroplasty	32.5	7.09	*0.7895*
	Single-stage	50.5	13.08	*0.0787*
	Two-stage	43.6	8.91	*0.1006*
Single-stage		50.5	13.08	
	Resection arthroplasty	32.5	7.09	*0.0214*
	Two-stage	43.6	8.91	*0.3764*
Two-stage		43.6	8.91	
	Resection arthroplasty	32.5	7.09	*0.0465*
		Difference in constant score^a^		
		Mean	SD	
Two-stage		30.5	14.85	
	Resection arthroplasty^b^	14.00	-	*-*
	Single-stage	21.50	13.44	*0.5901*
	Permanent Spacer^b^	12.00	-	*-*

* Statistically significant if *p* < 0.05

^a^ Determined by post-operative Constant score minus pre-operative scores

^b^ Only 1 paper per group, therefore no SD score or *p*-value

## Discussion

This systematic review demonstrates that the number of studies reporting two-stage exchange arthroplasty for established periprosthetic shoulder infection is approximately double that of each other treatment modality. However, we were unable to show a statistical difference in the eradication rate between the various treatments under study (*p = 0.650*); moreover, while we observed statistically better mean pre- and post-operative Constant scores in patients treated with a single-stage exchange, it was not possible to demonstrate a statistically significant improvement of shoulder function when comparing post- to pre-operative values of different treatment modalities.

The failure to demonstrate a superiority of one treatment in eradicating infection differs from recently reported systematic reviews regarding established hip and knee periprosthetic infections. Romano et al. demonstrated a superiority of two-stage exchange for the treatment of periprosthetic total knee infection compared to a single-stage exchange [17], which was also evident following a two-stage exchange for periprosthetic hip infection [22]. The present study included a much lower number of published papers and reported cases of infected shoulder prosthesis compared to the aforementioned systematic reviews. The relatively low number of patients reported may explain, at least in part, the inability to demonstrate a statistical difference between infection eradication rates.

Another potential bias of our analysis of the data is patient’s selection in the different studies considered. In this regard, it should be noted that the vast majority of studies failed to describe the indications for their treatments. We can only speculate that specific host and pathogen characteristics influenced the surgeon to perform a specific operation over another, but on review of the study cohorts, we did not find data to support a difference in age, severity of infection, type of microorganism or duration of infection in patients treated according to different treatment modalities. This may suggest that the choice largely relies on a surgeon or hospital-based routine, rather than on an established protocol and consistent case-by-case evaluation.

Similarly, it is unclear from the included studies the type of antibiotic regime that is adopted as part of their pre- and post-operative management, and only 44 % of these studies documented the length of the antibiotic treatment [Table 2]. The combination of appropriate targeted antibacterial therapy based upon confirmed cultures; together with an initial radical wound debridement is paramount to eradicating the infection [23]. No standard antibiotic regime exists and varies widely [24], but the importance of this omission in some studies must be appreciated.

We did not identify any publications relating to eradication and functional rates following these procedures using national joint registry (NJR) data. We believe the data from NJR is fundamentally flawed for the surveillance of infection procedures, as it is currently impossible to identify which treatment modality has the highest infection eradication rate, or has the best post-operative functional outcome as the British NJR, for example, seeks to collect pre-operative Oxford functional scores but not post-operative scores [25]. We believe the adoption of the NJR in this way will be extremely useful and may be fundamental in guiding future treatment.

Furthermore, patients treated with a single-stage procedure had, in the few studies that reported this data, a better average pre-operative function than those receiving a two-stage revision. Similar findings regarding pre-operative function were retrieved when comparing single-stage revision with permanent spacer or resection arthroplasty, in the only paper that reported those values [21]. If we assume pre-operative joint function as a rough indicator of the overall status of the patient, we may speculate that according to these findings, patients treated with a single-stage procedure may have had a less severe condition that those treated with a two-stage exchange or other treatment modalities.

Concerning more closely functional results, although a single-stage exchange arthroplasty would theoretically be associated with a functional advantage, compared to a permanent spacer or a resection arthroplasty, we did not find data to support this hypothesis, due to the lack of pre-operative data. However, a two-stage exchange may be hindered by the soft tissue insult, in particular rotator cuff insufficiency, as a result of the initial aggressive debridement and subsequent re-implantation at a later stage [14, 26], even if the presence of the interim spacer permits constant expansion of the soft tissues [3]; however, when comparing pre- and post-operative Constant scores, the difference was not statistically different when comparing single- and two-stage exchange. Coste et al. [21] noted from their series that greater preservation of function occurred with shorter delays in diagnosis and definitive treatment.

In conclusion, if surgical aims are to eradicate infection and prevent recurrence, whilst optimizing the function of the joint [14], the result of this systematic review failed to dictate when one treatment should be used over another; moreover, it should be noted that the surgical decision in a given patient relies also on a number of other variables, that were not analyzed in this review.

Furthermore, we acknowledge the following limitations of this study, which reflects the quality of the included studies and the available information:

- Omission of patient-related variables that may directly influence outcome; including patient co-morbidities, ASA grade, number of previous shoulder procedures, indication for primary procedure;
- Omission of surgeon-related variables that may directly influence outcome; including type of implant, type of spacer, use of antibiotic-impregnated cement, duration and use of antibiotics, quality and timing of initial debridement;
- No subgroup analysis comparing the type of antibiotic spacers (custom-made, molded or preformed), time interval between stages of a two-stage exchange, use of antibiotic-loaded cement and respective dose and type used
- No subgroup analysis comparing the outcome based upon the primary procedure (i.e. proximal humeral fracture, osteoarthritis, rotator cuff arthropathy, rheumatoid arthritis, or avascular necrosis) as the clinical course of infections may be very different in these subgroups;
- Outcome measures did not include quality of life scores, complication rates or aseptic revision rate;
- Inclusion of very few studies that directly compare changes in Constant scores, and therefore demonstrate an improvement or worsening in their patient cohort. It is well known that good pre-operative function is a predictor of good post-operative function, likewise pre-operative stiffness is a negative predictor [14]
- Inclusion of only papers published with either an abstract or full text in English
- Socioeconomic impact for each treatment

Also, we did not distinguish between recurrent and new infections, as this distinction was not made in most of the papers. The criteria for differentiating between recurrent and new infections is weakly supported in the literature, and somewhat artificial, and we believe this distinction is unreliable. The results of cultural examination in periprosthetic infection are too unpredictable, especially after previous antibiotic treatments.

Our findings should still be regarded as preliminary, since sample sizes are small and further confirmation is required when more data becomes available for review. Clearly there is a need for a large, multi-center, prospective study to establish the superiority of one surgical treatment over another.

## Conclusions

This systematic review failed to demonstrate a clear difference in infection eradication between a single- or two-stage exchange arthroplasty, use of permanent spacer, or resection arthroplasty, for established periprosthetic shoulder infection. Moreover, functional improvements, poorly reported in the majority of studies, were not shown to be significantly different between treatment modalities.

## Abbreviations

PJI: Periprosthetic Joint Infection
SD: Standard Deviation
N: Number
P: Probability
